# Combination of Fat-Free Muscle Index and Total Spontaneous Portosystemic Shunt Area Identifies High-Risk Cirrhosis Patients

**DOI:** 10.3389/fmed.2022.831005

**Published:** 2022-04-12

**Authors:** Anton Faron, Jasmin Abu-Omar, Johannes Chang, Nina Böhling, Alois Martin Sprinkart, Ulrike Attenberger, Jürgen K. Rockstroh, Andreas Minh Luu, Christian Jansen, Christian P. Strassburg, Jonel Trebicka, Julian Luetkens, Michael Praktiknjo

**Affiliations:** ^1^Department of Radiology, University Hospital Bonn, Bonn, Germany; ^2^Department of Internal Medicine I, University Hospital Bonn, Bonn, Germany; ^3^Department of Surgery, St. Josef Hospital, Ruhr-University Bochum, Bochum, Germany; ^4^Department of Internal Medicine I, University of Frankfurt, Frankfurt, Germany; ^5^European Foundation for the Study of Chronic Liver Failure, Barcelona, Spain

**Keywords:** sarcopenia, cirrhosis, spontaneous portosystemic shunt, fat-free muscle index, hepatic encephalopathy, acute decompensation, acute-on-chronic liver failure, ACLF

## Abstract

**Background:**

Sarcopenia and spontaneous portosystemic shunts (SPSSs) are common complications of liver cirrhosis, and both are associated with higher rates of hepatic encephalopathy (HE) development in these patients. This study aimed to evaluate the simultaneous impact of skeletal muscle mass and spontaneous portosystemic shunting, measured from routine diagnostic CT on outcomes in patients with liver cirrhosis.

**Methods:**

Retrospective analysis of patients with cirrhosis. Skeletal muscle mass [including fat-free muscle index (FFMI) as a surrogate for sarcopenia] and total cross-sectional spontaneous portosystemic shunt area (TSA) were quantified from CT scans. The primary endpoint was the development of HE, while the secondary endpoint was 1-year mortality.

**Results:**

One hundred fifty-six patients with liver cirrhosis were included. Patients with low (L-) FFMI and large (L-)TSA showed higher rates of HE development. In multivariable analysis, L-FFMI and L-TSA were independent predictors of HE development (L-FFMI HR = 2.69, CI 1.22–5.93; L-TSA, HR = 2.50, CI = 1.24–4.72) and 1-year mortality (L-FFMI, HR = 7.68, CI 1.75–33.74; L-TSA, HR = 3.05, CI 1.32–7.04). The simultaneous presence of L-FFMI and L-TSA exponentially increased the risk of HE development (HR 12.79, CI 2.93–55.86) and 1-year mortality (HR 13.66, CI 1.75–106.50). An easy sequential algorithm including FFMI and TSA identified patients with good, intermediate, and poor prognoses.

**Conclusion:**

This study indicates synergy between low skeletal muscle mass and large TSA to predict exponentially increased risk of HE development and mortality in liver cirrhosis. Simultaneous screening for sarcopenia and TSA from routine diagnostic CT may help to improve the identification of high-risk patients using an easy-to-apply algorithm.

**Clinical Trial registration:**

[ClinicalTrials.gov], identifier [NCT03584204].

## Introduction

Liver cirrhosis is a major health care burden, particularly due to its variety of severe complications caused by portal hypertension, such as variceal bleeding, ascites, and hepatic encephalopathy (HE), leading to high hospitalization rates and increased morbidity and mortality in these patients (1).

Portal hypertension is known to precipitate the development of spontaneous portosystemic shunts (SPSSs), which is frequently found in advanced stages of liver cirrhosis. Interestingly, a recently large international study showed that, although the prevalence of portosystemic shunts increased with deteriorating liver function, the presence of portosystemic shunts was associated with an increased risk for complications and also death in patients with preserved liver function (2). Accordingly, in another report, the TSA as a quantitative measure of portosystemic shunting was shown to predict HE and mortality development, independent of liver function (3).

Another increasingly recognized complication of liver cirrhosis is a continuous decline of skeletal muscle mass and function, commonly termed sarcopenia, which was shown to be frequent among patients with decompensated stages of disease (4, 5). Recently, an increasing number of studies demonstrated its negative impact on the outcome, especially with respect to the development of HE, waitlist mortality, and overall survival (5–11). This has led to the inclusion of sarcopenia in the current nutrition guidelines of the European Association of the Study of the Liver (EASL) (12).

In this context, it has been suggested that portosystemic shunting may directly contribute to muscle wasting (12, 13). Circulating blood can bypass the hepatic perfusion *via* collaterals, which may lead to increased ammonia levels in skeletal muscles and has been suggested to mediate myocyte autophagy. Moreover, muscular ammonia metabolism is known to deplete amino acids, which are crucial for the maintenance of muscle cells (14). Both skeletal muscles and portosystemic shunts can be reliably quantified from routine cross-sectional imaging and, hence, may be used to determine sarcopenia and the amount of portosystemic shunting, respectively (15).

However, the clinical interplay of these conditions, as well as their joint impact on the outcome in patients with liver cirrhosis, is not fully understood yet. Hence, this study aimed to explore (I) the synergetic impact of sarcopenia and portosystemic shunting on outcomes in patients with liver cirrhosis and (II) to determine whether quantification of these parameters from routine diagnostic imaging may help to improve the risk stratification for deleterious outcomes in these patients.

## Materials and Methods

### Study Population

**FIGURE 1:**
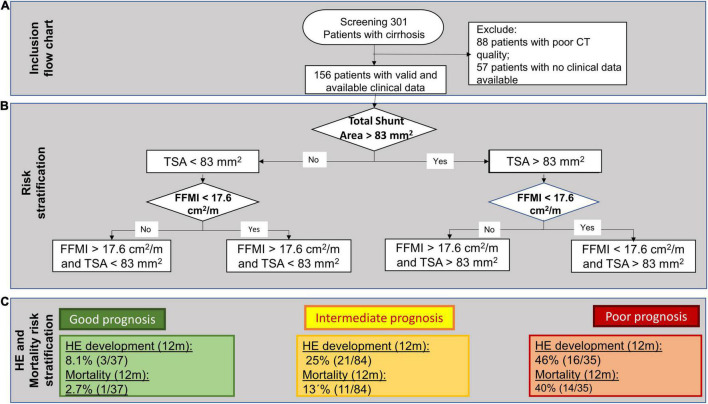
**(A)** A flowchart of patient inclusion. **(B)** A decision tree algorithm for hepatic encephalopathy (HE) development and mortality, sequentially, including total spontaneous portosystemic shunt (SPSS) Area (TSA) and Fat-Free Muscle Index (FFMI). **(C)** Prognostic groups and their respective rates of 1-year HE development and 1-year mortality.

For this study, consecutive patients, who presented to our centre from 2010 through 2015 due to liver cirrhosis, were retrospectively evaluated (Figure 1A). The included patients were at least 18 years old. Diagnosis of liver cirrhosis was made by clinical, histological, or imaging criteria. Patients were excluded if no diagnostic CT scan was available or if the image quality precluded an adequate assessment of portosystemic shunts and skeletal muscle mass. The baseline was set at the time of the CT scan. The clinical data and laboratory parameters were reviewed for baseline and a follow-up period of 1 year.

The primary endpoint was the development of HE (assessed by West-Haven criteria and neuropsychometric tests) and the secondary endpoint was 1-year survival. The study was performed in accordance with the Declaration of Helsinki. The study was approved by the institutional review board, and the necessity for written informed consent was waived due to its retrospective and monocentric character (ClinicalTrials.gov Identifier: NCT03584204).

### Assessment of TSA

Radiologists with an expertise in abdominal diagnostic imaging screened available CT scans [all with an indication for Hepatocellular Carcinoma (HCC) screening] for the presence of SPSS. Portosystemic shunts were identified as additional vessels originating from the superior and inferior mesenteric vein, the splenic vein, the portal vein, the renal veins, and the inferior vena cava and were verified from sagittal and coronal reformations. As it was reported previously, the largest short-axis diameter of the relevant shunt vessel was measured to obtain the maximal vessel diameter and to calculate the TSA (3).

As it was done in previous studies, gastric, esophageal, and anal varices were excluded from the calculation of TSA, as these are rather considered vessel networks and, therefore, do not allow for exact determination of vessel diameter and, thereby, vessel area (16).

### Assessment of Fat-Free Muscle Index

All patients underwent routine diagnostic multislice CT imaging of the abdomen in a supine position with the administration of iodinated contrast on a clinical CT-scanner (iCT, Philips Healthcare, Amsterdam, Netherlands). The typical imaging parameters were slice thickness of 1 or 2 mm, tube current (exposure time product) of 100 mAs, and tube voltage of 120 kVp.

Skeletal muscle areas of the paraspinal skeletal muscles at the intervertebral disc space level, between the third and fourth lumbar vertebra, were previously demonstrated to be highly correlated with total compartment volume and, therefore, were used for the estimation of skeletal muscle mass in this study (15).

To determine muscle quality, the skeletal muscle area was separated into areas of fatty and lean muscles based on densitometric thresholds. Fatty and lean muscle tissues were identified by ranges of low [–30 to 29 Hounsfield units (HU)] and high attenuation (30–100 HU), respectively. Skeletal muscle index (SMI) was measured as proposed in a previous study (12). Moreover, fat-free muscle area (FFMA) was calculated for each patient and was normalized for the patient’s height to obtain fat-free muscle index (FFMI) using the equation:


(1) $$\text{FFMI}=\text{FFMA}\left[{\text{cm}}^{2}\right]/\mathrm{height}\left[\text{m}\right].$$

### Statistical Analysis

We performed descriptive statistics for all variables. A non-parametric testing was used to compare different groups when suitable. The correlation of metric variables was performed using Spearman’s rank correlation coefficient. For the selection of cut-off values to determine low and high FFMI, a receiver-operating characteristics (ROC) analysis with the development of HE within a 1-year follow-up was calculated using the Youden index. The cut-off for TSA was used as previously reported in a large multicentre cohort (3).

The Kaplan–Meier analysis with the log-rank test was used to determine the impact of TSA and FFMI/SMI on the development of HE and mortality. Univariate and multivariate risk analyses were performed, including factors with the potential impact of outcome [age, baseline laboratory values, history of HE episodes, Chronic-liver-failure Consortium Acute Decompensation score (CLIF-C AD), as well as measurements of portosystemic shunt area and sarcopenia] with the Cox regression for 1-year mortality and occurrence of HE. A multivariate analysis included all values with *p* < 0.05 from univariate Cox regression. Prognostic scores with overlapping parameters (CLIF-C AD, MELD, and Child-Pugh score) were not entered simultaneously in multivariate regression analyses due to collinearity. The number of liver transplantation (LT) as competing events was low (8%). Thus, LT was censored, and competing risk analysis was not performed.

Continuous variables are presented as median (range). Categorical variables are presented as absolute cases or percentages. All data were analysed using statistics software SPSS (version 25, IBM, Armonk, NY, United States). The *p-*value < 0.05 was considered a statistically significant difference.

## Results

### General Patient Characteristics

Of the 301 evaluated patients, automated muscle measurement was not possible in 88 patients, which were therefore excluded. Of the remaining 213 patients, a clinical follow-up was available in 156 patients (Figure 1A). In this cohort, the median age at baseline was 58 (31–85) years and 92 (59%) patients were male. The majority had alcoholic cirrhosis (82, 53%). Thirty-one (21%) patients had viral liver cirrhosis and 43 (28%) had other causes of cirrhosis.

Seventy-eight patients (53%) had a history of ascites, 47 (30%) had gastrointestinal bleeding, and 21 (14%) had reported prior episodes of HE. At baseline, 92 (59%) patients exhibited ascites and 36 (23%) had an episode of HE. Seventeen patients (11%) were diagnosed with hepatic cellular carcinoma (within Milan criteria) at baseline.

At baseline, most patients had decompensated liver cirrhosis according to the Child-Pugh classification (55% with Child-Pugh class B/C). The median MELD and CLIF-C-AD scores were 12 (6–40) and 47 (31–78), respectively. Further general characteristics are detailed in Table 1.

**TABLE 1:** General characteristics stratified for 1-year hepatic encephalopathy (HE) development.

	Parameter median (range) or absolute (%)	All (*n* = 156)	Patients without 1-year HE development (*n* = 116)	Patients with 1-year HE development (*n* = 40)
Baseline General	Age (in years)	58 (31–85)	57 (31–85)	62 (39–79)**
	Sex (male/-female)	92/64 (59/41%)	70/40 (64/36%)	22/24 (48/52%)*
	Etiology of cirrhosis (alcoholic/viral/others)	82/31/43 (53/20/28%)	65/22/29 (56/19/25%)	17/9/14 (43/23/35%)
	Height (in m)	1.72 (1.5–1.92)	1.73 (1.52–1.9)	1.70 (1.5–1.92)
	Weight (in kg)	77 (39–147)	78 (49–147)	79 (39–110)
Historical Clinical Events	Ascites	78 (53%)	49 (42%)	29 (72%)**
	Hepatocellular carcinoma	17 (11%)	14 (12%)	3 (8%)
	Hepatic encephalopathy	21 (14%)	12 (10%)	9 (23%)
	Spontaneous bacterial peritonitis	9 (6%)	6 (6%)	3 (8%)
	Hepatorenal syndrome	19 (12%)	12 (10%)	7 (18%)
	Gastrointestinal bleeding	47 (30%)	39 (34%)	8 (21%)
Baseline Clinical Events	Ascites	92 (59%)	64 (55%)	28 (70%)
	Hepatic encephalopathy	36 (23%)	22 (19%)	14 (35%)**
	Spontaneous bacterial peritonitis	16 (10%)	10 (9%)	6 (15%)
	Hepatorenal syndrome	22 (14%)	14 (12%)	8 (20%)
	Gastrointestinal bleeding	18 (12%)	16 (14%)	2 (7%)
Baseline Scores	MELD	12 (6–40)	11 (6–33)	13 (6–24)*
	MELD-Na	13 (6–40)	12 (6–33)	14 (7–28)
	Child-Pugh score	7 (5–13)	6 (5–13)	7 (5–10)**
	Child-Pugh (class A/B/C)	65/69/12 (45/47/8%)	54/45/9 (50/42/8%)	11/24/3 (29/63/8%)**
	CLIF-C-AD	47 (31–78)	45 (31–78)	49 (37–71)**
Baseline Laboratory	Sodium (mmol/l)	138 (122–147)	138 (122–147)	138 (127–144)
	Creatinine (mg/dl)	0.9 (0.3–5.1)	0.9 (0.3–3.3)	1.1 (0.6–5.1)
	Bilirubin (mg/dl)	1.7 (0.2–34.8)	1.5 (0.2–12)	1.8 (0.2–13.1)
	AST (U/l)	50 (12–387)	48 (12–300)	56 (14–190)
	ALT (U/l)	32 (8–282)	33 (8–187)	32 (11–282)
	Albumin (g/l)	31 (3–49)	32 (3–45)	29 (3–44)*
	INR	1.2 (0.9–3)	1.2 (0.9–3)	1.2 (1–2.4)
	WBC (10^3^/μl)	5.7 (1–35.1)	5.2 (1.6–35.1)	5.7 (1.5–18.8)
	CRP (mg/dl)	9.5 (0.2–172)	7.9 (0.2–172)	11.4 (0.9–148)
	Platelets (×10^9^/L)	111 (24–440)	109 (29–440)	126 (36–272)

*p < 0.05, **p < 0.01, ***p < 0.001.

*MELD(-Na) Score, Model of End-Stage Liver Disease (Natrium) Score; CLIF-C-AD, chronic-liver-failure Consortium Acute Decompensation Score; AST, aspartat transaminase; ALT, alanine transaminase; INR, internationale normalized ratio (of prothrombin time); WBC, white blood cells; CRP, C-reactive protein.*

Median follow-up was 19 (0–97) months. Within 1-year follow-up, 40 patients (26%) developed at least one episode of HE. These patients were significantly older, were predominantly female, and showed worse prognostic scores (Table 1). Compared to the 116 patients who did not develop HE in the 1-year follow-up, they were more likely to have ascites in their prior clinical history (ascites: 72 vs. 42%, *p* = 0.001) and HE at baseline (HE: 35 vs. 19%; *p* = 0.002). Moreover, baseline serum albumin levels were significantly lower in patients who experienced episodes of HE (29 g/l vs. 32 g/l, *p* = 0.014, Table 1).

### Sarcopenia and TSA Classification

The mean FFMI was significantly lower in patients who developed episodes of HE compared to patients without episodes of HE within the follow-up period (24.8 vs. 32.1 cm^2^/m, *p* = 0.042). With the receiver operating characteristics (ROC) analysis, with an HE development within 1-year follow-up as an outcome, an area under the curve (AUC) of 0.623 (*p* = 0.023, CI 0.522–0.724) was observed for FFMI. The optimal cut-off value was found at 17.6 cm^2^/m (sensitivity 78%, specificity 47%) *via* the Youden index. Analysing sex-specific cut-offs did not improve performance. Therefore, sarcopenia was defined by a cut-off value of 17.6 cm^2^/m with patients having a lower FFMI classified as being sarcopenic (L-FFMI). In total, 96 (62%) patients were defined as sarcopenic and 60 (38%) as not sarcopenic.

To quantify the amount of portosystemic shunting, a previously validated cut-off value was used with patients having a TSA above 83 mm^2^ defined as having large TSA (L-TSA) (3).

### Association of Sarcopenia and TSA With HE and Mortality

**FIGURE 2:**
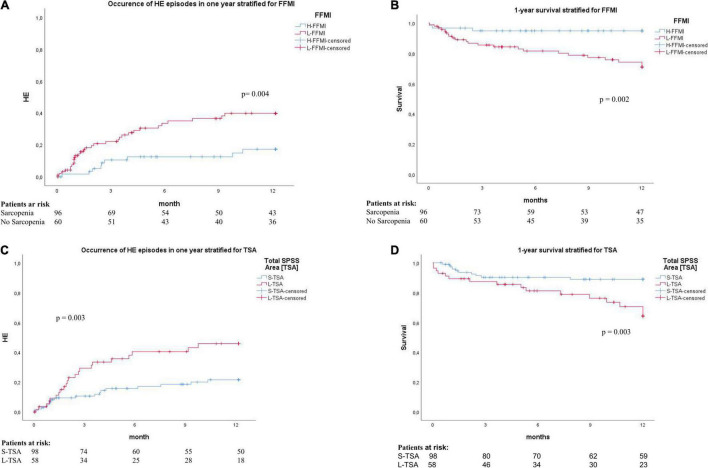
**(A)** Cumulative incidence of development HE in the 1-year follow-up, stratified by FFMI. H-FFMI (high FFMI, blue line); L-FFMI (low FFMI, red line). P by log-rank. **(B)** Kaplan–Meier survival plot for 1-year survival, stratified by FFMI. H-FFMI (high FFMI, blue line); L-FFMI (low FFMI, red line). P by log-rank. **(C)** Cumulative incidence of development HE in the 1-year follow-up, stratified by TSA. S-TSA (small TSA, blue line); L-TSA (large TSA, red line). P by log-rank. **(D)** Kaplan-Meier survival plot for 1-year survival, stratified by TSA. S-TSA (small TSA, blue line); L-TSA (large TSA, red line). P by log-rank.

The Kaplan-Meier analysis showed that patients with sarcopenia exhibited significantly higher rates of HE development (32 vs. 15%, *p* = 0.004) and a higher 1-year mortality (24 vs. 5%, *p* = 0.002) (Figures 2A,B). Also, the patients with L-TSA were more likely to develop episodes of HE (45 vs. 30%, *p* = 0.003) and showed significantly higher 1-year mortality (31 vs. 20%, *p* = 0.003) (Figures 2C,D).

Acute-on-chronic liver failure (ACLF) was the most common cause of death (89%). Only three patients (11%) died of other causes (all malignancy) within 12 months follow-up (Supplementary Table 1). Of note, a patient stratification by L3-SMI (gender-specific cut-off values of 38.5 kg/m^2^ for women and 52.4 kg/m^2^ as validated in previous studies) did not show a significant difference for the development of HE and a 1-year mortality in this cohort (Supplementary Figures 1A,B). Therefore, L3-SMI was not used for further stratification.

To identify risk factors for the occurrence of HE, risk factor stratifications using Cox-regression analyses were performed. In a multivariate analysis, including all factors that were significantly associated with HE and 1-year mortality on the respective univariate analysis, large TSA, low FFMI, history of HE episodes, and CLIF-C AD remained as independent predictors for developments of HE within 1-year follow-up (Table 2). Similarly, large TSA, low FFMI, and CLIF-C AD were independent predictors of 1-year mortality (Table 3). Patients with sarcopenia (low FFMI) exhibited a distinctly higher risk to develop episodes of HE [hazard ratio (HR) = 2.685, 95% CI 1.215–5.932] and showed markedly increased risk of 1-year mortality (HR = 7.683, 95% CI 1.749–33.743). Similarly, individuals with L-TSA exhibited a higher risk to develop HE (HR 2.500, 95% CI 1.324–4.718) and to die within this time period (HR 3.050, 95% CI 1.323–7.035).

**TABLE 2:** Univariate/multivariate Cox regression analysis for 1-year HE-development.

Parameters	univariate Cox-Regression				multivariate Cox-Regression			
	p	HR	CI Lower	CI Upper	p	HR	CI Lower	CI Upper
L-FFMI	0.006	2.809	1.336	3.908	0.015	2.685	1.215	5.932
L-TSA	0.005	2.460	1.318	4.591	0.005	2.500	1.324	4.718
Previous HE	<0.001	1.848	1.344	2.542	<0.001	2.001	1.425	2.809
CLIF-C-AD	0.003	1.050	1.017	1.085	0.023	1.039	1.005	1.073
MELD	0.042	1.049	1.002	1.098				
Child-Pugh	0.007	1.221	1.056	1.413				
Age	0.007	1.048	1.013	1.084	0.090			
Bilirubin	0.034	1.132	1.009	1.269	0.402			
Platelets	0.690	0.999	0.996	1.003				
CRP	0.444	1.004	0.993	1.016				

*L-FFMI, low fat-free muscle index; L-TSA, large total shunt area; CLIF-C-AD, chronic-liver-failure Consortium Acute Decompensation Score; MELD, model for end-stage liver disease; CRP, C-reactive protein.*

**TABLE 3:** Univariate/multivariate Cox regression analysis for 1-year mortality.

Parameters	univariate Cox-Regression				multivariate Cox-Regression			
	P	HR	CI Lower	CI Upper	p	HR	CI Lower	CI Upper
L-FFMI	0.006	5.460	1.639	18.191	0.007	7.683	1.749	33.743
L-TSA	0.005	3.069	1.391	6.772	0.009	3.050	1.323	7.035
CLIF-C-AD	<0.001	1.083	1.045	1.122	0.004	1.061	1.019	1.105
MELD	<0.001	1.149	1.087	1.215				
Child-Pugh	<0.001	1.724	1.414	2.102				
Age	0.062	1.040	0.998	1.083				
Bilirubin	<0.001	1.168	1.084	1.259	0.023	1.144	1.018	1.285
Platelets	0.211	0.997	0.992	1.002				
CRP	0.004	1.014	1.004	1.023	0.096			

*L-FFMI, low fat-free muscle Index; L-TSA, large total shunt area; CLIF-C-AD, chronic-liver-failure Consortium Acute Decompensation Score; MELD, model for end-stage liver disease; CRP, C-reactive protein.*

### Combination of FFMI and TSA for the Prediction of HE Development and Mortality

To assess dependency between muscle mass (FFMI) and TSA, we performed a correlation analysis. This showed no significant correlation of FFMI with TSA (Supplementary Figure 2).

Using a decision tree algorithm, sequentially including FFMI and TSA, an easy to assess prognostic algorithm was developed (Figure 1B). First, the CT scans are assessed for the presence of L-TSA and, then, further stratified by the presence of L-FFMI. With this algorithm, patients were stratified into three risk groups: good, intermediate, and poor prognoses (Figure 1C).

**FIGURE 3:**
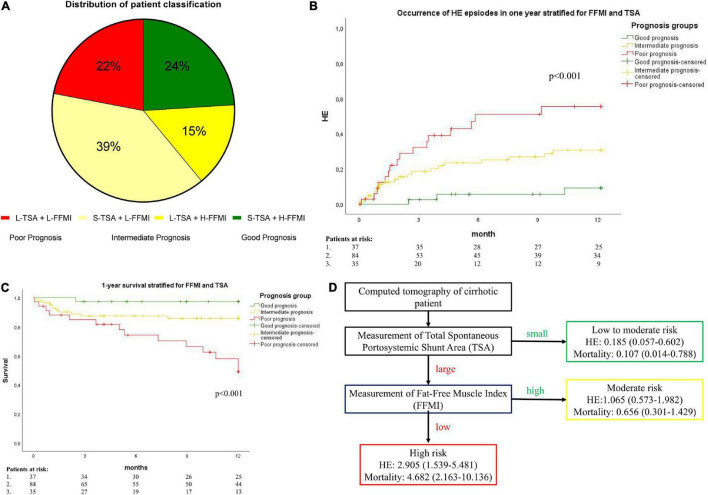
**(A)** A pie diagram of patient distribution according to TSA and FFMI. H-FFMI (high FFMI); L-FFMI (low FFMI); S-TSA (small TSA); L-TSA (large TSA). **(B)** Cumulative incidence of the development of HE, and **(C)** A Kaplan–Meier survival analysis stratified by prognostic groups. The good prognosis [H-FFMI + S-TSA (small TSA); green line]; intermediate prognosis (L-FFMI + S-TSA and H-FFMI + L-TSA; yellow line); poor prognosis (L-FFMI + L-TSA; red line). P by log-rank. **(D)** Flow diagram of risk of hepatic encephalopathy (HE) and overall mortality stratified by TSA and FFMI. Hazard ratio and 95%-confidence interval are shown.

Accordingly, the cohort was subdivided into these three subsets [Good prognosis: high FFMI and small TSA, *n* = 37 (24%); Intermediate prognosis: high FFMI and large TSA, *n* = 23 (15%) plus low FFMI and small TSA, *n* = 61 (39%); Poor prognosis: low FFMI and large TSA, *n* = 35 (22%); Figure 3A]. In the Kaplan–Meier analysis, comparing these subcohorts, the highest and lowest rates of HE development was observed for poor and good prognoses groups, respectively (Figure 3B). This result was confirmed in competing risk analysis for HE, with death and LT as competing events (Supplementary Figure 3). Similarly, the poor prognosis group had the highest 1-year mortality, while the good prognosis group had the lowest (Figure 3C).

According to these data, additional risk factor analyses for the development of HE and mortality were performed, comparing good and poor prognoses groups. In a multivariate Cox regression analysis, the simultaneous presence of L-FFMI and L-TSA (poor prognosis group), alongside the history of HE episodes, was the only independent predictor for the development of HE within 1-year follow-up (HR 12.790, 95% CI 2.928–55.864, *p* = 0.001, Table 4). Regarding 1-year mortality, the simultaneous presence of L-FFMI and L-TSA (poor prognosis group, HR 13.660, 95% CI 1.752–106.495, *p* = 0.013), CLIF-C-AD (HR 1.085, 95% CI 1.019–1.155, *p* = 0.011) were independent predictors (Table 5 and Figure 3D). These data suggest an exponentially increased risk of the development of HE and of mortality in patients with L-FFMI and L-TSA.

**TABLE 4:** Univariate/multivariate Cox regression analysis for 1-year HE development comparing lower fat-free muscle index (L-FFMI) and large-total portosystemic shunt area(L-TSA) classification with high fat-free muscle index (H-FFMI) and small-TSA (S-TSA) classification.

1-year HE development	univariate Cox-Regression				multivariate Cox-Regression			
	*p*	HR	CI Lower	CI Upper	*p*	HR	CI Lower	CI Upper
L-FFMI & L-TSA	<0.001	9.013	2.615	31.064	0.001	12.790	2.928	55.864
Previous HE	<0.001	1.848	1.344	2.542	0.006	1.842	1.194	2.841
CLIF-C-AD	0.003	1.050	1.017	1.085	0.086			
Age	0.007	1.048	1.013	1.084	0.180			
Bilirubin	0.034	1.132	1.009	1.269	0.184			
CRP	0.444	1.004	0.993	1.016				

*L-FFMI, low fat-free muscle Index; H-FFMI, high fat free muscle index; S-TSA, small total shunt area; L-TSA, large total shunt area; CLIF-C-AD, chronic-liver-failure Consortium Acute Decompensation Score; CRP, C-reactive protein.*

**TABLE 5:** Univariate/multivariate Cox-Regression for 1-year mortality comparing L-FFMI and L-TSA classification with H-FFMI and S-TSA classification.

1-year mortality	univariate Cox-Regression				multivariate Cox-Regression			
	*p*	HR	CI Lower	CI Upper	*p*	HR	CI Lower	CI Upper
L-FFMI and L-TSA	0.004	20.312	2.666	154.774	0.013	13.660	1.752	106.495
CLIF-C-AD	<0.001	1.083	1.045	1.122	0.011	1.085	1.019	1.155
Age	0.062	1.040	0.998	1.083				
Bilirubin	<0.001	1.168	1.084	1.259	0.110	1.172	0.965	1.424
CRP	0.004	1.014	1.004	1.023	0.350			

*L-FFMI, low fat-free muscle Index; H-FFMI, high fat free muscle index; S-TSA, small total shunt area; L-TSA, large total shunt area; CLIF-C-AD, chronic-liver-failure Consortium Acute Decompensation Score; CRP, C-reactive protein.*

## Discussion

The present study describes the interplay of sarcopenia and TSA/SPSS in patients with liver cirrhosis. The L-FFMI and L-TSA as measures of sarcopenia and large portosystemic shunting, respectively, were identified as independent predictors for deleterious outcomes in patients with liver cirrhosis (3, 8, 10). Notably, these factors were observed not to be interrelated with one another in our cohort. If, however, both L-FFMI and L-TSA were present simultaneously, the risk for the development of HE and mortality within 1-year follow-up increases exponentially, independent of liver function. As both factors can be easily quantified from routine diagnostic CT-imaging, they may represent promising new imaging biomarkers for outcome stratification. In this context, an easy-to-apply prognostic algorithm is proposed by this study to complement the established methods of risk stratification.

Sarcopenia is generally accepted as a major risk factor for worsened outcomes in chronic diseases (16–18). As it was also shown to be related to adverse outcomes in patients with liver cirrhosis, it was recently included in international guidelines such as the Clinical Practice Guidelines of the European Association for the Study of the Liver (EASL) (12). Several studies dealt with the association of sarcopenia with clinical outcomes and mortality in patients with cirrhosis. One study showed that patients with sarcopenia were more likely to have a minimal HE and a higher risk of developing an episode of overt HE (OHE) (8). In other studies, sarcopenia was shown to predict complication rates and waiting-list mortality in patients prior to liver transplantation (9, 19). Studies on the outcomes following liver transplantation evaluated pre-transplant sarcopenia and showed poorer 1-year survival rates for sarcopenic recipients of liver transplants (11).

A CT-derived assessment of muscle areas is based on densitometric thresholds. Therefore, particularly in patients with liver disease, a distortion of anatomical composition and, thereby, precision of body compartment measurements due to fluid overload is a real concern. To mitigate the impact of ascites on the accuracy of muscle measurements, we decided to quantify skeletal muscles from the paraspinal compartment, as this site is distant from the abdominal cavity and was recently shown to allow for the estimation of skeletal muscle mass (15). Moreover, previous studies indicated that myosteatosis is an independent risk factor for HE development (20) and that, beyond mere muscle mass, a fat-free muscle fraction as another indicator of muscle quality seems to be of prognostic value, particularly in patients with liver disease (5, 10, 21, 22). Hence, the lean muscle fraction normalized for body height as an objective and comparable indicator of muscle quality, which can be opportunistically derived from diagnostic imaging, was investigated in this study.

Spontaneous portosystemic shunting is not only a risk factor for poor clinical outcomes on its own (2, 3). Moreover, patients with SPSS in combination with transjugular intrahepatic portosystemic shunt (TIPS) have been shown to have a higher risk of developing episodes of HE (23). However, the interplay of SPSS with other risk factors has not been studied, so far. In the presented study, the prognostic value of SPSS/TSA was complemented by adding FFMI, opportunistically measured from the same CT scans.

Particularly in patients with decompensated liver cirrhosis, adequate risk stratification and identification of high-risk patients (24, 25) are crucial due to a plethora of severe resulting complications.

Among the available risk factors, sarcopenia and portosystemic shunting particularly appeared suitable for evaluation as these factors were not only shown to predict severe complications and increased mortality but also represent potential therapeutic targets (26–28). Shunt embolization was shown to significantly decrease the risk of developing OHE (23, 29), while amelioration of sarcopenia prior to TIPS was demonstrated to enhance the clinical outcome, and a muscular activity has been shown to improve portal hypertension (10, 30, 31). Importantly, previous episodes of HE could be confirmed as a strong independent predictor of HE development and, thus, underlines the robustness of our data.

Although some previous studies indicated a potential interrelation between portosystemic shunts and sarcopenia (2, 13), these factors were observed to predict fatal outcomes independent from one another in liver cirrhosis, as well as independent from liver function in our study. According to the survival analysis conducted in our study, both factors seemed to contribute to an increased risk for HE development at a similar rate; the risk exponentially increased when both factors were present simultaneously. This observation may indicate a potentially synergetic impact on the outcome for these factors and, therefore, may imply an additional predictive value by simultaneous measurement. It should be pointed out that this study does not aim to replace existing algorithms for sarcopenia screening and treatment, like the one presented in the current EASL Clinical Practice Guidelines (12), but rather complements them by adding SPSS/TSA as another aspect that can be easily measured opportunistically from the same scans.

The present study, therefore, warrants active screening for both sarcopenia and portosystemic shunting in patients with liver cirrhosis for several reasons. First, screening for these factors may help to facilitate the identification of high-risk patients, who may require intensified monitoring. Beyond that, early detection of sarcopenia and relevant portosystemic shunting ensure punctual therapeutic interventions and may contribute to a more precise and individualized treatment approach in these patients. A diagnostic CT is performed for several indications in patients with liver cirrhosis, such as evaluation for liver transplantation, TIPS, or hepatocellular carcinoma. Here, both sarcopenia and portosystemic shunts may be opportunistically quantified from available imaging and do not require additional efforts for assessment.

We acknowledge several limitations of this study. As with other monocentric retrospective investigations, the generality of the observation cannot be warranted, and validation studies are needed. Additionally, the impact of etiology could not be explored due to the small sample size and the diverse cohort, even though risk factors for sarcopenia and shunting vary depending on the cause of cirrhosis. Our results should be validated to define the relevance in specific etiologies.

Moreover, the retrospective character precluded functional assessment of muscle function. However, our results indicate that both sarcopenia and portosystemic shunting, which are frequent among patients with liver cirrhosis in various stages of the disease, may have a substantial impact on outcomes in these patients, independent from liver function. As these factors can be easily quantified from routine diagnostic CT imaging, our findings, therefore, are legitimately larger and have especially prospective investigations, which ultimately may reinforce the utility of our findings for clinical routine.

Due to the retrospective design, a selection bias cannot be ruled out. The reasons for patient exclusion were mainly missing follow-up data or poor image quality. Also, even though the main characteristics did not differ significantly between the included and the excluded patients, those who were eliminated from the study had significantly higher MELD- and Child-Pugh-Scores, as well as slightly less alcoholic liver disease. Therefore, our findings need further validation, especially in patients with advanced liver disease, but this was beyond the scope of our study.

It should also be noted that despite the presented prognostic value of our algorithm, liver function is still the main predictor of clinical outcomes, including HE development and mortality. Due to the lack of a validation cohort, we were not able to establish and properly calibrate a combined score like the MELD-sarcopenia score (32). This could be researched in future.

In conclusion, this study may indicate a synergistic impact of sarcopenia and portosystemic shunting on the outcome with an exponential risk increase for HE development and mortality, when both factors are present. Underlining the strength of our data, the role of sarcopenia and portosystemic shunting as biomarkers for deleterious outcomes in patients with liver cirrhosis was confirmed. This may suggest a great value of opportunistic screening for both sarcopenia and portosystemic shunts, from a routine diagnostic CT in patients with liver cirrhosis, in identifying high-risk patients with an easy-to-apply prognostic algorithm.

## Data Availability Statement

The datasets presented in this article are not readily available because the dataset is restricted by GDPR. Requests to access the datasets should be directed to MP, michael.praktiknjo@ukbonn.de.

## Ethics Statement

The studies involving human participants were reviewed and approved by Ethikkommission der Medizinischen Fakultät der Universität Bonn. Written informed consent for participation was not required for this study in accordance with the national legislation and the institutional requirements.

## Author Contributions

AF and JA-O: acquisition of data, analysis and interpretation of data, drafting of the manuscript, and statistical analysis. JC, NB, AS, and CJ: acquisition of data, analysis and interpretation of data. UA, AL, JR, and JT: administrative support, interpretation of data, and critical revision of the manuscript regarding important intellectual content. JL and MP: study concept and design, acquisition of data, analysis and interpretation of data, drafting of the manuscript, critical revision of the manuscript regarding important intellectual content, funding recipient, administrative, technical and material support, and study supervision. All authors contributed to the article and approved the submitted version.

## Conflict of Interest

The authors declare that the research was conducted in the absence of any commercial or financial relationships that could be construed as a potential conflict of interest.

## Publisher’s Note

All claims expressed in this article are solely those of the authors and do not necessarily represent those of their affiliated organizations, or those of the publisher, the editors and the reviewers. Any product that may be evaluated in this article, or claim that may be made by its manufacturer, is not guaranteed or endorsed by the publisher.

## Supplementary Material

Supplementary Figure 1 **(A)** Cumulative incidence of development HE in the 1-year follow-up, stratified by L3-SMI. No sarcopenia (blue line); sarcopenia (red line). P by log-rank. **(B)** Kaplan–Meier survival plot for 1-year survival, stratified by L3-SMI. No sarcopenia (blue line); sarcopenia (red line). P by log-rank.

Supplementary Figure 2 Correlation plot for FFMI and TSA.

Supplementary Figure 3 Competing risk analysis for HE and death/liver transplantation (LT).

## Abbreviations

(O)HE: (overt) hepatic encephalopathy
HRS: hepatorenal syndrome
ACLF: acute-on-chronic liver failure
CLIF-C: European Foundation for the study of chronic liver failure consortium
AD: acute decompensation
TPMT: transversal psoas muscle thickness
MRI: magnetic resonance imaging
CT: computed tomography
ROC: receiver operating characteristics
AUC: area under the curve
HU: Hounsfield unit
MELD: model of end-stage liver disease
INR: international normalized ratio
WBC: white blood cell count
HR: hazard ratio
95% CI: 95% confidence interval
EASL: European Association of the Study of the Liver
SMI: skeletal muscle index
LT: liver transplantation
FFMI: Fat-free muscle index
SPSS: spontaneous portosystemic shunt.
