# Supplementary figures and images for: Combination of Fat-Free Muscle Index and Total Spontaneous Portosystemic Shunt Area Identifies High-Risk Cirrhosis Patients
Source: Front Med (Lausanne). 2022 Apr 12;9:831005. doi: 10.3389/fmed.2022.831005 (PMC9040492; doi:10.3389/fmed.2022.831005)

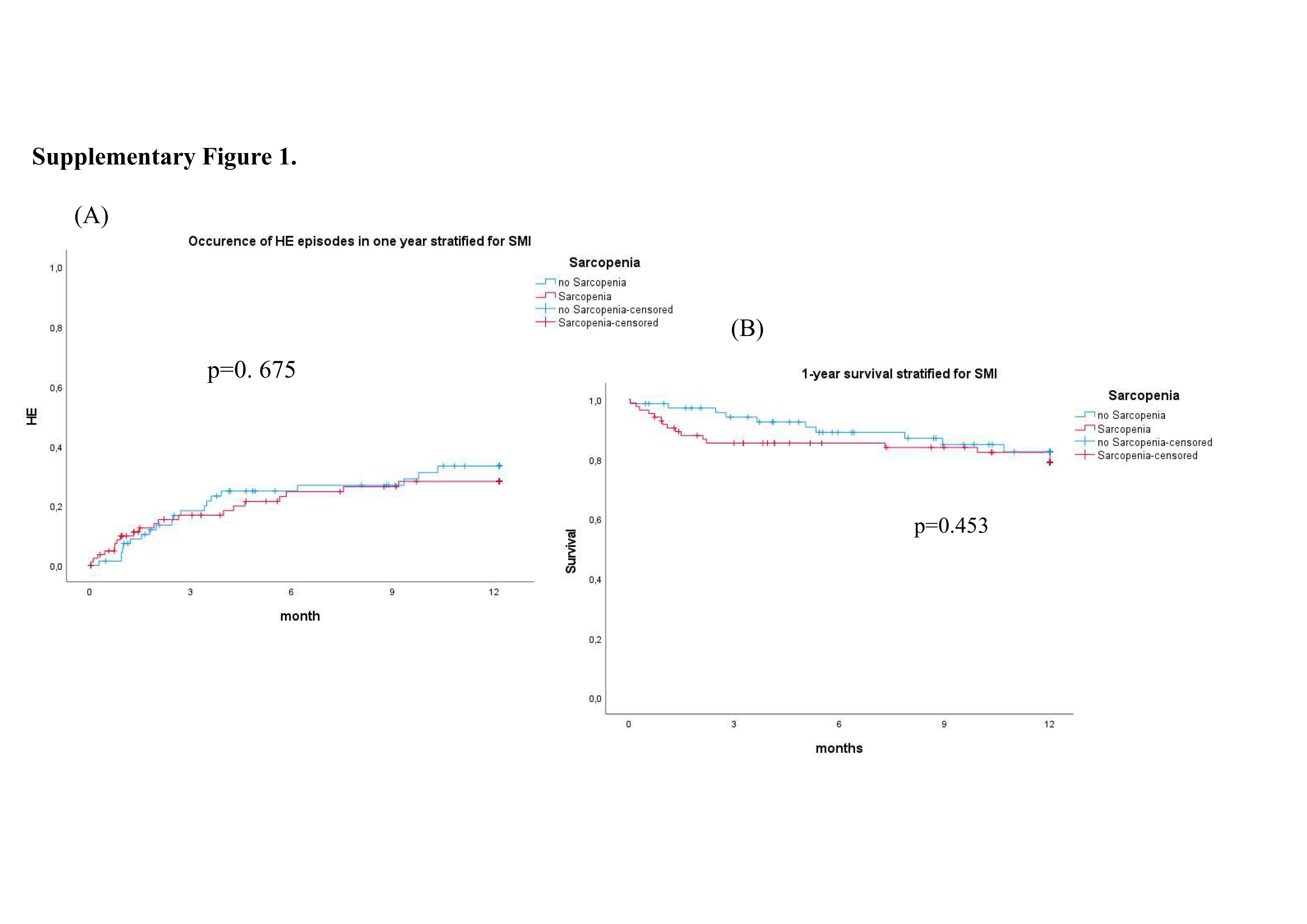

Supplement: Supplementary Figure 1 — (A) Cumulative incidence of development HE in the 1-year follow-up, stratified by L3-SMI. No sarcopenia (blue line); sarcopenia (red line). P by log-rank. (B) Kaplan–Meier survival plot for 1-year survival, stratified by L3-SMI. No sarcopenia (blue line); sarcopenia (red line). P by log-rank. [file Image_1.TIF]

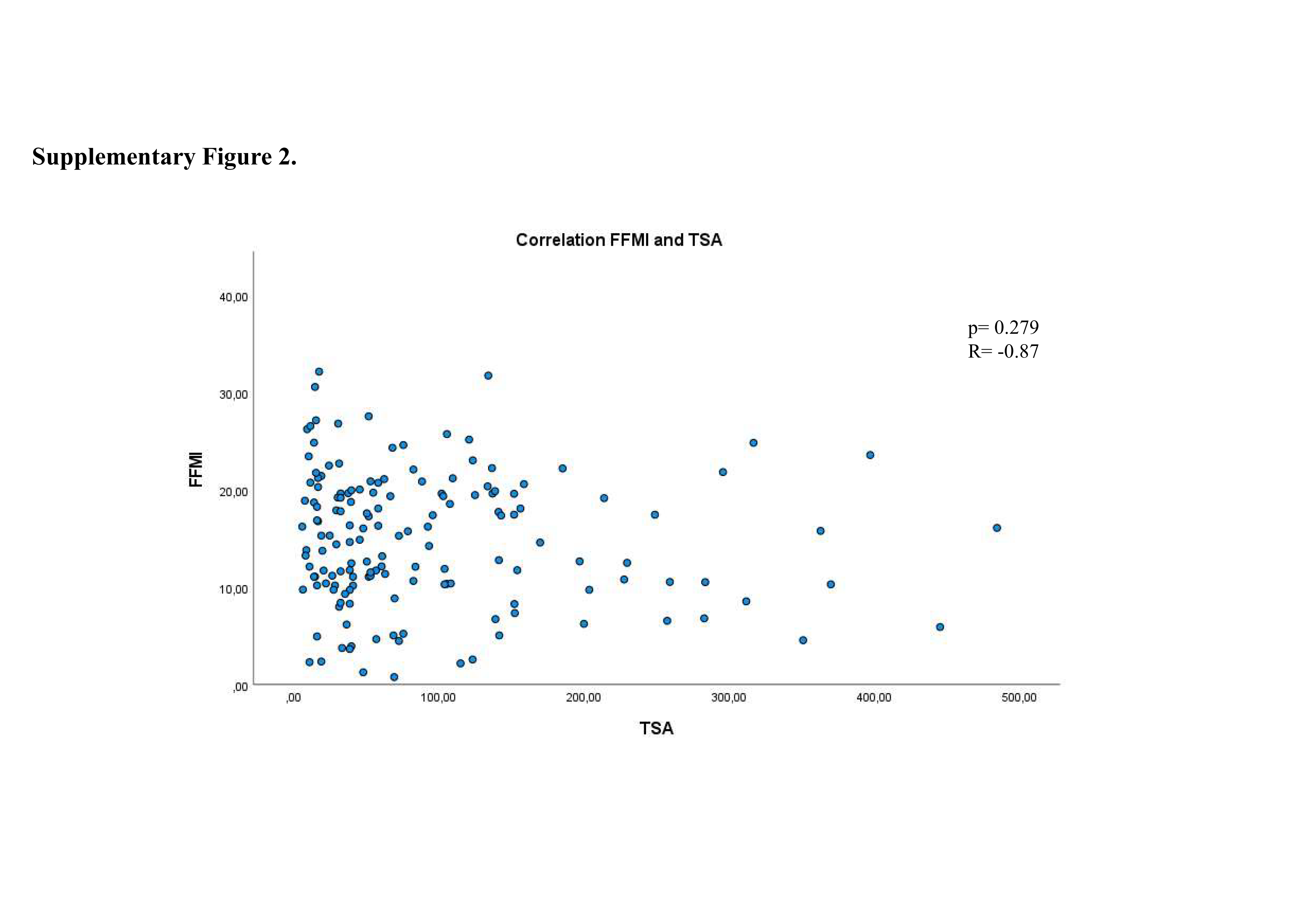

Supplement: Supplementary Figure 2 — Correlation plot for FFMI and TSA. [file Image_2.TIF]

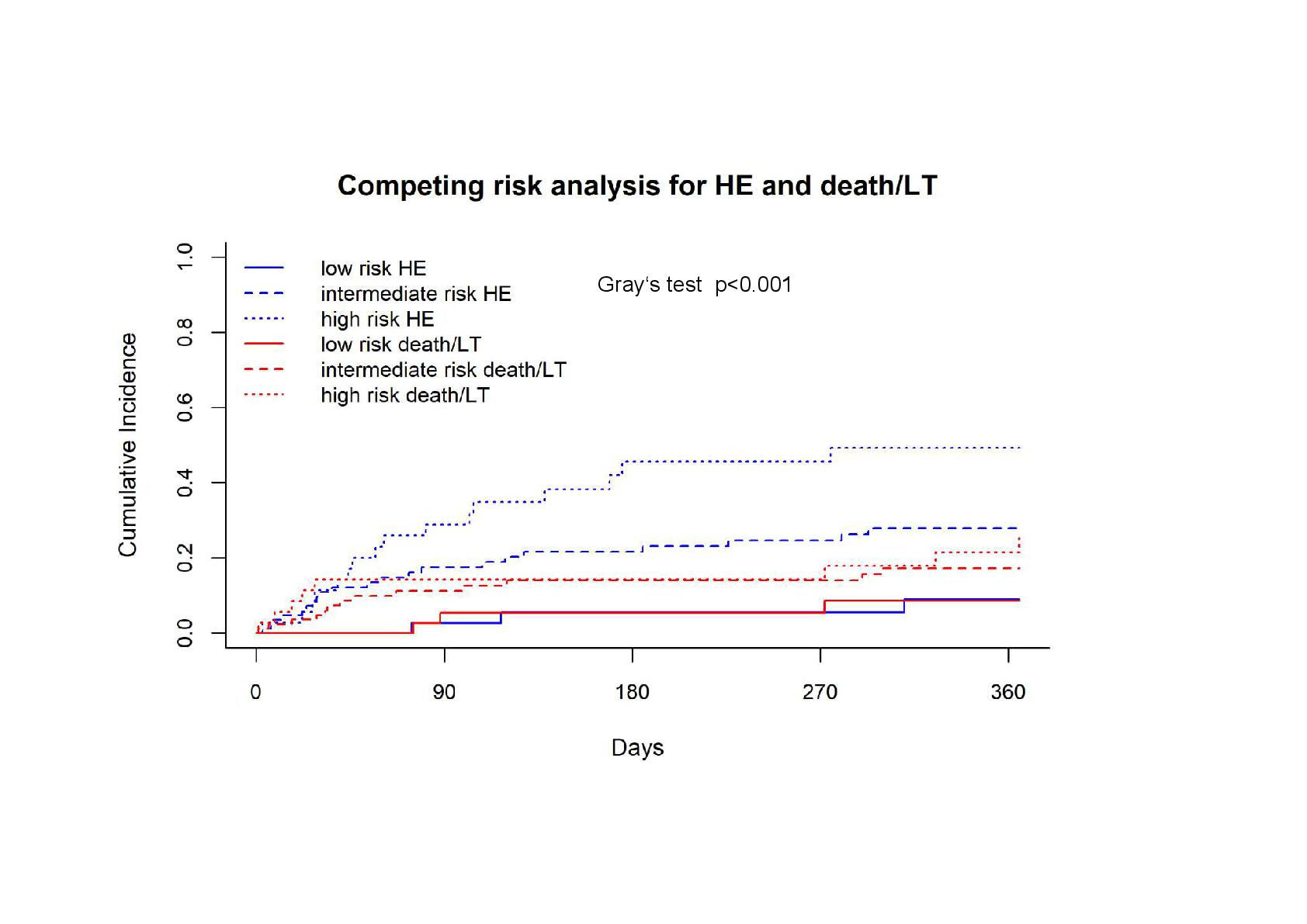

Supplement: Supplementary Figure 3 — Competing risk analysis for HE and death/liver transplantation (LT). [file Image_3.tiff]
