# Supplementary material for: Combination of Fat-Free Muscle Index and Total Spontaneous Portosystemic Shunt Area Identifies High-Risk Cirrhosis Patients
Source: Front Med (Lausanne). 2022 Apr 12;9:831005. doi: 10.3389/fmed.2022.831005 (PMC9040492; doi:10.3389/fmed.2022.831005)
Supplement: Supplementary file 4 [file Table_1.DOCX]

**Supplementary Table 1.: Causes of death during 1-year follow up stratified by prognosis groups.**

| **Causes of death** | **Good prognosis** | **Intermediate prognosis** | **Poor prognosis** |
| --- | --- | --- | --- |
| Acute-on-chronic liver failure | 1 (100%) | 10 (91%) | 12 (86%) |
| Malignancy | 0 (0%) | 1 (9%) | 2 (14%) |
| Other | 0 (0%) | 0 (0%) | 0 (0%) |
