# Supplementary material for: Combination of Fat-Free Muscle Index and Total Spontaneous Portosystemic Shunt Area Identifies High-Risk Cirrhosis Patients
Source: Front Med (Lausanne). 2022 Apr 12;9:831005. doi: 10.3389/fmed.2022.831005 (PMC9040492; doi:10.3389/fmed.2022.831005)
Supplement: Supplementary file 5 [file Table_2.docx]

**Supplementary Table 2.: General Characteristics of excluded patients**

|  | **Parameter median (range) or absolute (%)** | **all**  **(n=145)** |
| --- | --- | --- |
| **Baseline General** | Age (in years) | 57 (28-84) |
|  | Sex (male/-female) | 77/68 (53/47%) |
|  | Etiology of cirrhosis (alcoholic/viral/others) | 92/29/25 (63/20/17%) * |
|  | Height (in m) | 1.73 (1.51-1.98) |
|  | Weight (in kg) | 84 (41-140) * |
| **Historical Clinical Events** | Ascites | 65 (49%) |
|  | Hepatocellular carcinoma | 13 (9%) |
|  | Hepatic encephalopathy | 26 (19%) |
|  | Spontaneous bacterial peritonitis | 11 (8%) |
|  | Hepatorenal syndrome | 11 (8%) |
|  | Gastrointestinal bleeding | 38 (29%) |
| **Baseline Clinical Events** | Ascites | 102 (70%) * |
|  | Hepatic encephalopathy | 42 (29%) |
|  | Spontaneous bacterial peritonitis | 16 (11%) |
|  | Hepatorenal syndrome | 27 (19%) |
|  | Gastrointestinal bleeding | 30 (21%) ** |
| **Baseline Scores** | MELD | 14 (6-39) ** |
|  | MELD-Na | 16 (6-39) ** |
|  | Child-Pugh score | 7 (5-13) ** |
|  | Child-Pugh (class A / B / C) | 38/74/22 (28/55/16%) ** |
|  | CLIF-C-AD | 49 (23-85) * |
| **Baseline Laboratory** | Sodium [mmol/l] | 138 (119-154) |
|  | Creatinine [mg/dl] | 1 (0.4-6) |
|  | Bilirubin [mg/dl] | 2.2 (0.4-48) * |
|  | AST [U/l] | 53 (15-653) |
|  | ALT [U/l] | 28 (8-349) |
|  | Albumin [g/l] | 31 (3-49) |
|  | INR | 1.3 (0.9-4.6) ** |
|  | WBC [10³/µl] | 6.6 (1.3-37.2) * |
|  | CRP (mg/dl) | 14.8 (1-160) *** |
|  | Platelets [x10^9^/L] | 98 (11-653) |

*p < 0.05, ** p < 0.01, *** p < 0.001 vs. included cohort

MELD(-Na) Score: Model of End-Stage Liver Disease (Natrium) Score

CLIF-C-AD: Chronic-liver-failure Consortium Acute Decompensation Score

AST: Aspartat transaminase

ALT: Alanine transaminase

INR: Internationale normalized ratio (of prothrombin time)

WBC: White blood cells

CRP: C-reactive protein
